# Early laparoscopic cholecystectomy after percutaneous transhepatic gallbladder drainage for acute cholecystitis

**DOI:** 10.1038/s41598-021-82089-4

**Published:** 2021-01-28

**Authors:** Yunxiao Lyu, Ting Li, Bin Wang, Yunxiao Cheng

**Affiliations:** 1 Department of Hepatobiliary Surgery, Affiliated Dongyang Hospital of Wenzhou Medical University, 60 West Wuning Road, Dongyang, 322100 Zhejiang People’s Republic of China; 2 Department of Personnel Office, Affiliated Dongyang Hospital of Wenzhou Medical University, Dongyang, 322100 Zhejiang People’s Republic of China

**Keywords:** Gall bladder, Gall bladder

## Abstract

There is no consensus on the optimal timing of laparoscopic cholecystectomy (LC) after percutaneous transhepatic gallbladder drainage (PTGBD) for patients with acute cholecystitis (AC). We retrospectively evaluated patients who underwent LC after PTGBD between 1 February 2016 and 1 February 2020. We divided patients into three groups according to the interval time between PTGBD and LC as follows: Group I (within 1 week), (Group II, 1 week to 1 month), and Group III (> 1 month) and analyzed patients’ perioperative outcomes. We enrolled 100 patients in this study (Group I, n = 22; Group II, n = 30; Group III, n = 48). We found no significant difference between the groups regarding patients’ baseline characteristics and no significant difference regarding operation time and estimated blood loss (*p* = 0.69, *p* = 0.26, respectively). The incidence of conversion to open cholecystectomy was similar in the three groups (*p* = 0.37), and we found no significant difference regarding postoperative complications (*p* = 0.987). Group I had shorter total hospital stays and medical costs (*p* = 0.005, *p* < 0.001, respectively) vs Group II and Group III. Early LC within 1 week after PTGBD is safe and effective, with comparable intraoperative outcomes, postoperative complications, and conversion rates to open cholecystectomy. Furthermore, early LC could decrease postoperative length of hospital stay and medical costs.

## Introduction

Laparoscopic cholecystectomy (LC) has now replaced open cholecystectomy (OC) as the first choice for cholecystectomy, with the advantages of less pain, shorter hospital stay, and shorter recovery time. However, debate continues regarding whether LC is beneficial for acute cholecystitis (AC)^1, 2^, even though several studies showed that LC is safe and effective for AC^3, 4^. For patients with AC, LC is recommended soon after onset if there are no contradictions for the procedure. However, the treatment benefit depends on the AC severity and whether the patient can tolerate emergency surgery. Percutaneous transhepatic gallbladder drainage (PTGBD) was first reported in the early 1980s and was recommended in several guidelines to manage patients with AC with a high risk for LC^1, 5^. PTGBD is beneficial for improving patients’ conditions to permit delayed LC^6^; however, the optimal time for LC after PTGBD is controversial, and few studies have provided quality evidence. Several papers have discussed the interval time between LC and PTGBD; however, these studies revealed mixed results regarding perioperative outcomes^7–9^. Sakamoto et al. performed a nationwide database study; however, OC was included in this study^7^. The Tokyo guidelines updated in 2018 revealed that there was no consensus regarding the optimal timing of LC after PTGBD^1^, and, related to various factors, it is still very difficult to perform randomized controlled studies. In the current study, we retrospectively investigated the optimal timing of LC after PTGBD.

## Results

### Baseline characteristics

We divided the 100 patients into three groups: 22 patients in Group I (14 men and 8 women); 30 patients in Group II (16 men and 14 women); and 48 patients in Group III (26 men and 22 women). The mean age of the patients was 65.41 ± 15.43 years (Group I), 68.81 ± 17.36 years (Group II), and 66.71 ± 12.69 years (Group III). Patients’ baseline characteristics appear in Table 1. There were no significant differences in age, sex, American Society of Anesthesiologists score, Charlson comorbidity index, and laboratory data between the three groups.

**Table 1:** The baseline characteristics of included patients.

	Group I (n = 22)	Group II (n = 30)	Group III (n = 48)	*P*
Age, years	65.4 (3.29)	68.81 (3.17)	66.71 (1.83)	0.699
Sex (male)	14	16	26	0.263
Time to PTGBD, days	3.45 (0.28)	3.18 (0.28)	2.59 (1.83)	0.199
Time intervals between PTGBD and LC, days	5.28 (0.27)	22.93 (1.33)	52.19 (5.09)	< 0.001
BMI, kg/m^2^	23.28 (0.81)	23.5 (0.56)	23.7 (0.50)	0.89
Temperature, °	37.38 (0.18)	37.5 (0.14)	37.42 (0.13)	0.876
WBC, 10^9^/L	12.84 (0.99)	14.8 (1.46)	13.19 (0.14)	0.507
PLT, 10^9^/L	147.17 (11.96)	156.77 (8.90)	161.73 (10.62)	0.672
CRP, mg/L	99.7 (11.90)	102.75 (13.53)	106.14 (10.62)	0.924
AST, U/L	150.83 (19.32)	186.71 (63.95)	100.17 (27.60)	0.284
ALT, U/L	120.5 (23.87)	267.71 (93.172)	158.91 (14.24)	0.151
ALP, U/L	120.67 (11.91)	113.28 (9.51)	126.73 (14.24)	0.817
Creatine, μmol/L	76.5 (4.82)	72.23 (6.17)	77.09 (17.07)	0.829
Combines comorbid	10 (45.45)	17 (56.67)	24 (50.00)	0.713
**Cardiovascular disease**	6 (27.27)	9 (30.00)	10 (20.83)	
Diabetes mellitus	1 (4.55)	3 (10.00)	4 (8.33)	
COPD	1 (4.55)	2 (6.67)	3 (6.25)	
Chronic renal failure	1 (4.55)	0 (0)	2 (4.17)	
MT	0 (0)	2 (6.67)	2 (4.17)	
Others	1 (4.55)	1 (3.33)	3 (6.25)	
ASA (I/II/III/IV/V)	3/9/7/3/0	5/9/10/6/0	6/14/16/12/0	0.929
CCI	3.32 (0.12)	3.18 (0.11)	3.28 (0.07)	0.617
**Severity of AC**				
Grade I	4 (18.18)	8 (26.67)	8 (16.67)	0.546
Grade II	17 (77.27)	22 (73.33)	38 (79.17)	0.837
Grade III	1 (4.55)	0 (0)	2 (4.17)	0.514
Previous abdominal surgery	3 (13.64)	2 (6.67)	5 (10.42)	0.701

Values were presented as mean ± SEM or n (%).

*LC* laparoscopic cholecystectomy, *PTGBD* percutaneous transhepatic gallbladder drainage, *BMI* body mass index, *WBC* white blood cell, *PLT* blood platelet, *CRP* C-reactive protein, *AST* aspartate aminotransferase, *ALT* alanine aminotransferase, *ALP* alkaline phosphatase, *COPD* chronic obstructive lung disease, *MT* malignant tumor, *ASA* American Society of Anesthesiologist, *CCI* Charlson Comorbidity Index.

### Outcomes during the interval time

Group II had higher rates of recurrences cholecystitis (p = 0.044, Group I vs Group II) (Table 2). Group II and Group III had higher rates catheter-related complications (p = 0.044, Group I vs Group II, respectively; and p = 0.039, Group I vs Group III, respectively) (Table 2).

**Table 2:** The outcomes in waiting time.

	Group I (n = 22)	Group II (n = 30)	Group III (n = 48)	*P*
Recurrent cholecystitis	0	2	9	*0.044*
**Catheter-related complication**	0	5 (16.67)	12 (25.00)	*0.039*
Catheter dislodgement	0	2 (6.67)	4 (8.33)	
Catheter occlusion	0	2 (6.67)	4 (8.33)	
Cellulitis	0	1 (3.33)	3 (6.25)	
Bile leak	0	0	1 (2.08)	

Values were presented n (%).

### Intraoperative outcomes

We found no significant differences between the three groups for operation time (*p* = 0.699) and estimated blood loss (*p* = 0.263) (Table 3). Twelve patients were converted to OC (two in Group I, two in Group II, and eight in Group III). The reasons for conversion to OC were showed in Table 3. There was no significant difference between the groups regarding conversion to OC (*p* = 0.373) (Table 3).

**Table 3:** Comparison of intraoperative outcomes.

	Group I (n = 22)	Group II (n = 30)	Group III (n = 48)	*P*
Operation time, min	92.16 (4.50)	89.26 (3.88)	99.38 (5.22)	0.699
Estimated blood loss, ml	82.15 (13.95)	76.29 (10.98)	89.16 (8.01)	0.263
**Conversion to OC, %**	2 (9.09)	2 (6.67)	8 (16.67)	0.373
Uncontrollable bleeding, %	1 (4.55)	0	1 (2.08)	
Adhesion around the gallbladder, %	0	1 (3.33)	4 (8.33)	
Undistection of the Calot’s triangle, %	1 (4.55)	1 (3.33)	2 (4.17)	
Others, %	0	0	1 (2.08)	

Values are presented as mean ± standard deviation or n(%).

*OC* open cholecystectomy.

### Postoperative outcomes

Postoperative complications occurred in three patients in Group I, four patients in Group II, and seven patients in Group III (*p* = 0.987) (Table 4). The patient who experienced bile duct injury underwent endoscopy, and bile duct wall injury was identified. This complication resolved with nasal bile duct drainage for 12 days. There were no deaths within 30 days of surgery. The total hospital stay was shorter and total costs were lower in Group I than in Group II (*p* = 0.005) and between Group I and Group III (p < 0.001). Postoperative hospital stay was similar between the groups (*p* = 0.744) (Table 4).

**Table 4:** Comparison of postoperative outcomes.

	Group I (n = 22)	Group II (n = 30)	Group III (n = 48)	*P*
Total hospital stays, days	9.19 (1.15)^a^	12.28 (1.27)	15.29 (1.17)^a^	0.005
Postoperative hospital stays,days	5.28 (0.70)	5.34 (0.54)	4.87 (0.39)	0.744
Patients’ medical cost (¥)	20,358.45 (76.31)^b^	22,873.29 (69.08)^c^	24,927.39 (66.29)^bc^	< 0.001
**Postoperative complications, %**	3 (13.63)	4 (13.33)	7 (14.58)	0.987
Bile duct injury	0	0	1 (2.08)	
Wound infection	1 (4.55)	1 (3.33)	2 (4.17)	
Abdominal abscess	1 (4.55)	0	0	
Common bile duct stones	1 (4.55)	2 (6.67)	1 (2.08)	
Abdominal hemorrhage	0	0	1 (2.08)	
Pneumonia	0	1 (3.33)	1 (2.08)	
Pulmonary emobolism	0	0	1 (2.08)	
Reoperation, %	0	0	1 (2.08)	0.578

Values were presented as mean ± SEM or n (%).

^a^*P*: 0.005; ^b,c^*p* < 0.001.

## Discussion

Early LC within 1 week after PTGBD is safe and effective, with comparable intraoperative outcomes, postoperative complications, and conversion rates to open cholecystectomy. Furthermore, early LC could decrease postoperative length of hospital stay and medical costs.

Previous studies, including several meta-analyses, have confirmed the safety and efficacy of early LC within 7 days for patients with AC^2, 10^. However, many patients cannot tolerate surgery, and delayed surgery is necessary to achieve improved patient status. Additionally, gallbladder drainage is recommended for high-surgical-risk patients with AC. Several procedures, including endoscopic and percutaneous techniques, have been reported for gallbladder drainage^11, 12^, and PTGBD is recommended to resolve acute inflammation in AC. PTGBD can be performed with B-ultrasound guidance and local anesthesia. Although several reports discuss endoscopic gallbladder drainage, PTGBD is a safer substitute for cholecystectomy in high-risk patients, and can be followed by LC when patients’ status improves. However, the optimal timing for LC after PTGBD is unclear.

LC remains a challenge in patients with AC because LC may increase the possibility of conversion to OC. The incidence of conversion to OC varies from 11 to 28%, compared with < 5% in LC for chronic cholecystitis^13–15^. Twelve patients in our study were converted to OC, which is a similar rate to that in previous reports. The main reason for conversion to OC was inflammation and adhesions around the gallbladder. In early AC, inflammation and edema play important roles in contributing to conversion to OC. With a long interval after PTGBD, adhesions around the gallbladder and secondary fibrosis increase the difficulty of LC. The incidence of conversion to OC in our study was relatively low, with no significant difference between the three groups. It is worth noting that the difficulty of LC depends to some extent on the surgeon’s experience. The surgeons in our study perform more than 100 LC procedures per year; therefore, we believe that the decisions to convert to OC were appropriate. Several factors such as BMI and CRP levels are considered risk factors for conversion to OC^16, 17^; however, BMI and CRP levels in our study did not differ significantly between the groups.

Several studies have discussed the safety of early LC for patients with AC. A meta-analysis of 15 randomized controlled trials showed that early LC was as safe and effective as delayed LC within 7 days^10^. However, many patients undergoing PTGBD have underlying diseases or gallbladder inflammation that makes their conditions more serious, and the incidence of postoperative complications may increase as a result. Chikamori et al. evaluated 31 patients scheduled for early LC following PTGBD and 12 patients undergoing delayed LC, and confirmed the safety of early LC^18^. However, other authors showed that early LC was associated with higher postoperative complication rates^13^. In our study, we found no intergroup differences regarding postoperative complication rates. Our results provide further evidence supporting the safety of early LC following PTGBD.

Another advantage of early LC is that it can resolve cholecystitis earlier, and avoid cholecystitis recurrence and a series of catheter-related complications during the interval period, as well as decreasing the total length of hospitalization and hospitalization costs. We confirmed these results, in our study.

We included patients with Grade III AC severity in our study, and LC was performed when these patients’ status improvement. However, the sample size in this group was small, and whether this affected our results is unknown.

Debate is on-going regarding a benefit related to operation time and estimated blood loss with early LC. Han et al. found that their early group had longer operation times after comparing LC at 72 h vs > 72 h following PTGBD^13^. Similar results were observed in the study by Choi et al.^19^. In contrast, including in our study, several studies revealed that early LC was comparable to delayed LC regarding operation time^9^. However, the definition of early and delayed surgery varied in previous studies.

Estimated blood loss is also considered an indicator of safety in early LC. In our study, the comparison of estimated blood loss revealed no significant difference between the three groups.

Total hospital stay was shorter, and medical costs were lower in our Group I than in Group II and Group III. This may be related to the different time delays to surgery requiring more waiting time and necessary examinations; however, postoperative hospital stay was similar between the three groups, which also supports the safety of early surgery.

Despite our positive findings, as a retrospective study, certain limitations are present such as selection bias. Also, there were 20% patients with Grade I and only 3 patients with Grade III in this cohort. There were many reasons such as anticoagulant drugs or the patients refused to perform cholecystectomy. Additionally, the sample size in this study was small, and no consensus exists regarding the optimal timing of LC following PTGBD. More high quality studies, especially randomized controlled trials, are required.

In conclusion, our study confirmed the safety and efficacy of early LC within 1 week following PTGBD. This approach resulted in shorter hospital stays and lower medical costs with comparable intraoperative and postoperative outcomes vs later LC. Considering the limitations of our study, more high-quality randomized controlled studies are needed to clarify the ideal timing of LC for AC.

## Methods

### Patients

This retrospective study was performed in a single center that diagnoses and treats > 600 patients with AC per year and performs > 500 LCs per year. This study was approved by the ethics committees of our hospital.

**Figure 1:**
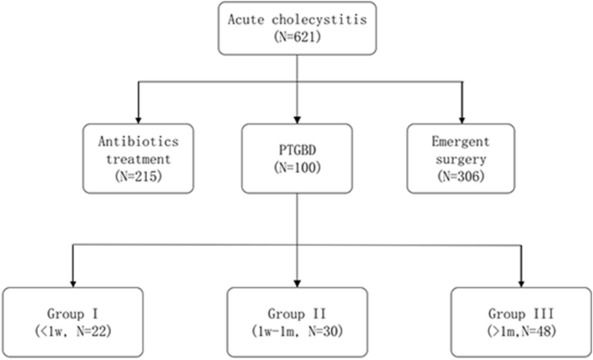
Study flow chart. PTGBD: percutaneous transhepatic gallbladder drainage.

We retrospectively reviewed the medical records of patients with AC treated between 1 February 2016 and 1 February 2020 (Fig. 1). We recorded patients’ age, sex, body mass index (BMI), comorbidities, and laboratory findings. We also recorded patients’ American Society of Anesthesiologists score and Charlson comorbidity index. The exclusion criteria were (1) patients who did not underwent LC after PTGBD; (2) patients with common bile duct stones; (3) patients with additional pancreatitis or cholangitis; (4) patients were lost to follow during the wait time after PTGBD. We divided the included patients into three groups according to the interval time between PTGBD and LC, as follows: Group I (< 1 week); Group II (1 week to 1 month); and Group III (> 1 month).

The diagnosis and severity of AC was made according to the Tokyo Guidelines^20^. All patients with AC were given intravenous antibiotherapy based on local bacterial epidemiology and germ sensitivity. At the same time, we provide appropriate liquid supplements. The patient could be discharged when the patient's abdominal pain is relieved, the body temperature is normal, and the WBC and CRP were normal. Patients were followed in the outpatients during the waiting time.

### PTGBD procedure

PTGBD was performed by two experienced ultrasonographer physicians who perform > 100 PTGBD procedures per year. PTGBD was performed under local anesthesia in an ultrasonography unit or in the intensive care unit. Using sterile technique with ultrasound guidance, an 8-Fr or 10-Fr self-locking pigtail catheter was placed via a transhepatic route. Post-procedure improvement was defined as resolution of symptoms and abnormal laboratory measurements.

### LC procedure

LC was performed by four experienced surgeons who perform > 100 LC procedures per year. Patients were placed in the supine position, and pneumoperitoneum was created with a Veress needle through a 10-mm subumbilical incision. A 30-degree optical instrument was placed through the subumbilical incision, and two additional working instruments were placed with the guidance of the optical instrument (10-mm subxiphoidal and 5-mm right subcostal along the midclavicular line). Gallbladder contents were aspirated in patients with gallbladder distension. Calot’s triangle was dissected first, and we attempted retrograde gallbladder dissection starting at the fundus in patients with severe inflammation and anatomical difficulty dissecting the pericystic space. The gallbladder was extracted through the subumbilical incision. Conversion to OC was performed, if necessary, as described previously. A right subcostal is made. It is important to obtain good visualization of the gallbladder, Triangle of Calot, and bile ducts. After clarifying the cystic duct, common hepatic duct, and common bile duct, the cystic duct and cystic artery are disconnected. The gallbladder then can be removed from the gallbladder bed of the liver using either electrocautery.

### Outcome measures

The outcome measures constituted outcomes during the interval time, intraoperative outcomes, and postoperative outcomes. Outcomes during the interval time were recurrent cholecystitis and catheter-related complications. Intraoperative outcomes were operation time, estimated blood loss, and conversion to OC, and postoperative outcomes were postoperative complications, total length of hospital stay, postoperative hospital stay, and medical costs. We scheduled telephone interviews or outpatient visits following PTGBD and LC.

### Statistical analysis

The data were analyzed using SPSS ver. 17.0 (SPSS Inc, Chicago, IL, USA). Descriptive statistics were calculated for demographic and clinical variables and were reported as means ± standard error mean (SEM). Comparisons between groups for categorical variables were assessed using the chi-square test. The Student t test was used to compare two groups for normally distributed quantitative variables otherwise Mann–Whitney test was used. Bonferroni test was used as a post hoc test for intergroup analysis. P < 0.05 was considered statistically significant. All authors had access to the study data and reviewed and approved the final article.

### Ethics

The Affiliated Dongyang Hospital of Wenzhou Medical University Institutional Review Board approved the protocol (No. 2020-YX-038) and all participants were provided written informed consent.

## References

[1] Okamoto K (2018). Tokyo guidelines 2018: Flowchart for the management of acute cholecystitis. J. Hepato Biliary Pancr. Sci..

[2] Vaccari S (2018). Early versus delayed approach in cholecystectomy after admission to an emergency department: A multicenter retrospective study. G Chir.

[3] Zafar SN (2015). Optimal time for early laparoscopic cholecystectomy for acute cholecystitis. JAMA Surg..

[4] Faizi KS, Ahmed I (2013). & Ahmad, H.

[5] Radder RW (1980). Ultrasonically guided percutaneous catheter drainage for gallbladder empyema. Diagn. Imaging.

[6] Sugiyama M, Tokuhara M, Atomi Y (1998). Is percutaneous cholecystostomy the optimal treatment for acute cholecystitis in the very elderly?. World J. Surg..

[7] Sakamoto, T., Fujiogi, M., Matsui, H., Fushimi, K. & Yasunaga, H. Timing of cholecystectomy after percutaneous transhepatic gallbladder drainage for acute cholecystitis: A nationwide inpatient database study. *Hpb* (2019). 10.1016/j.hpb.2019.10.2438 31732466

[8] Inoue, K.*, et al.* Optimal timing of cholecystectomy after percutaneous gallbladder drainage for severe cholecystitis. *BMC Gastroenterol.* **17**(2017). 10.1186/s12876-017-0631-8 PMC5452332 28569137

[9] Jung WH, Park DE (2015). Timing of cholecystectomy after percutaneous cholecystostomy for acute cholecystitis. Korean J. Gastroenterol..

[10] Lyu YX (2018). Same-admission versus delayed cholecystectomy for mild acute biliary pancreatitis: A systematic review and meta-analysis. BMC Surg..

[11] Krishnamoorthi R (2020). EUS-guided versus endoscopic transpapillary gallbladder drainage in high-risk surgical patients with acute cholecystitis: A systematic review and meta-analysis. Surg. Endosc..

[12] Ogura T, Higuchi K (2019). Endoscopic ultrasound-guided gallbladder drainage: Current status and future prospects. Dig. Endosc..

[13] Han IW (2012). Early versus delayed laparoscopic cholecystectomy after percutaneous transhepatic gallbladder drainage. J. Hepatobiliary. Pancreat. Sci..

[14] Abe K (2019). The efficacy of PTGBD for acute cholecystitis based on the Tokyo guidelines 2018. World J. Surg..

[15] Iino C (2018). Comparable efficacy of endoscopic transpapillary gallbladder drainage and percutaneous transhepatic gallbladder drainage in acute cholecystitis. Endosc. Int. Open.

[16] Asai K (2014). Risk factors for conversion of laparoscopic cholecystectomy to open surgery associated with the severity characteristics according to the Tokyo guidelines. Surg. Today.

[17] Sippey M (2015). Acute cholecystitis: Risk factors for conversion to an open procedure. J. Surg. Res..

[18] Chikamori F, Kuniyoshi N, Shibuya S, Takase Y (2002). Early scheduled laparoscopic cholecystectomy following percutaneous transhepatic gallbladder drainage for patients with acute cholecystitis. Surg. Endosc..

[19] Choi JW, Park SH, Choi SY, Kim HS, Kim TH (2012). Comparison of clinical result between early laparoscopic cholecystectomy and delayed laparoscopic cholecystectomy after percutaneous transhepatic gallbladder drainage for patients with complicated acute cholecystitis. Korean J. Hepato-Biliary-Pancreatic Surg..

[20] Yokoe M (2018). Tokyo Guidelines 2018: diagnostic criteria and severity grading of acute cholecystitis (with videos). J Hepatobiliary Pancreat Sci.

